# Homeostatic Changes in GABA and Acetylcholine Muscarinic Receptors on GABAergic Neurons in the Mesencephalic Reticular Formation following Sleep Deprivation

**DOI:** 10.1523/ENEURO.0269-17.2017

**Published:** 2018-01-03

**Authors:** Hanieh Toossi, Esther del Cid-Pellitero, Barbara E. Jones

**Affiliations:** Department of Neurology and Neurosurgery, Montreal Neurological Institute, McGill University, Montreal, Quebec H3A 2B4, Canada

**Keywords:** AChM2, GABAA, homeostasis, mice, waking

## Abstract

We have examined whether GABAergic neurons in the mesencephalic reticular formation (RFMes), which are believed to inhibit the neurons in the pons that generate paradoxical sleep (PS or REMS), are submitted to homeostatic regulation under conditions of sleep deprivation (SD) by enforced waking during the day in mice. Using immunofluorescence, we investigated first, by staining for c-Fos, whether GABAergic RFMes neurons are active during SD and then, by staining for receptors, whether their activity is associated with homeostatic changes in GABA_A_ or acetylcholine muscarinic type 2 (AChM2) receptors (Rs), which evoke inhibition. We found that a significantly greater proportion of the GABAergic neurons were positively stained for c-Fos after SD (∼27%) as compared to sleep control (SC; ∼1%) and sleep recovery (SR; ∼6%), suggesting that they were more active during waking with SD and less active or inactive during sleep with SC and SR. The density of GABA_A_Rs and AChM2Rs on the plasma membrane of the GABAergic neurons was significantly increased after SD and restored to control levels after SR. We conclude that the density of these receptors is increased on RFMes GABAergic neurons during presumed enhanced activity with SD and is restored to control levels during presumed lesser or inactivity with SR. Such increases in GABA_A_R and AChM2R with sleep deficits would be associated with increased susceptibility of the wake-active GABAergic neurons to inhibition from GABAergic and cholinergic sleep-active neurons and to thus permitting the onset of sleep and PS with muscle atonia.

## Significance Statement

Neuronal activity is regulated in a homeostatic manner such that prolonged activity results in increases in receptors to inhibitory neurotransmitters. Sleep-wake states are also regulated in a homeostatic manner likely through the regulation of sleep-wake regulatory neurons. Here, we established using c-Fos that GABAergic neurons in the mesencephalic reticular formation (RFMes), which are believed to inhibit pontine paradoxical sleep (PS or REMS) generating neurons, are active during enforced waking with sleep deprivation (SD). GABA_A_ and acetylcholine muscarinic type 2 receptors (AChM2Rs), which evoke inhibition, were increased on the membrane of the GABAergic neurons. The increase in these receptors would render the neurons more susceptible to inhibition by GABA and ACh and to thus permitting the onset of PS with muscle atonia.

## Introduction

Using transections and lesions, early studies established that the pontine tegmentum is critical for the generation of paradoxical sleep (PS or REMS) with muscle atonia (Jouvet, 1962). Using more localized lesions, subsequent studies indicated that the oral pontine (PnO) reticular formation (RF) was most critical (Carli and Zanchetti, 1965) including a zone extending laterally and dorsally into the subcoeruleus (SubC) and sublaterodorsal tegmental (SubLDT) nuclei (Sakai, 1980; Hendricks et al., 1982; Friedman and Jones, 1984; Webster and Jones, 1988; Lu et al., 2006). Injections of the cholinergic agonist, carbachol, into this region elicited PS, presumably through excitation of PS generating neurons (George et al., 1964; Gnadt and Pegram, 1986; Baghdoyan et al., 1987; Vanni-Mercier et al., 1989). Injections of glutamate agonists into the region even more potently elicited PS (Onoe and Sakai, 1995; Boissard et al., 2002). Moreover, neurons were identified there in immunohistochemical studies, which expressed c-Fos during PS rebound (Maloney et al., 1999, 2000) and in electrophysiological studies, which discharged during PS and are accordingly presumed to be PS-effector neurons (Sakai, 1980; Sakai and Koyama, 1996; Boucetta et al., 2014).

Multiple lines of evidence have indicated that PS-effector neurons in the pontine tegmentum are under inhibitory control through GABAergic input. Pharmacological studies showed that microinjection of bicuculline, a GABA_A_ receptor (GABA_A_R) antagonist, into the PnO and adjacent areas of the pontine tegmentum elicited PS with muscle atonia (Xi et al., 1999; Boissard et al., 2002; Sanford et al., 2003). Early lesion studies had indicated that lesions of neurons within the mesencephalic tegmentum, particularly in the ventrolateral periaqueductal gray (VLPAG) and adjoining mesencephalic RF (RFMes; also called deep mesencephalic nucleus (DpMe; Paxinos and Franklin, 2001) produced marked hypersomnia with a prominent increase in PS (Petitjean et al., 1975; Lu et al., 2006). A PS increase was also evoked by microinjection of muscimol, a GABA_A_R agonist, and conversely, a PS decrease was evoked by microinjection of bicuculline, a GABA_A_R antagonist, into this region (Sastre et al., 1996), suggesting that GABAergic neurons located in the VLPAG and RFMes could potentially inhibit the PS-effector neurons in the pons to which they project (Sapin et al., 2009). The most effective site was claimed to be within the RFMes (or dorsocaudal central tegmental field in the cat) where direct excitation by glutamate agonists also prevented PS and elicited waking (Crochet and Sakai, 1999). Indeed, GABAergic neurons are distributed through this field (Ford et al., 1995), and some expressed c-Fos with PS deprivation (Sapin et al., 2009). Thus, GABAergic neurons of the RFMes could possibly control the generation of PS by holding the PS-effector neurons in the pons under inhibition during waking. These neurons also appear to be controlled in turn by GABAergic inhibitory input through GABA_A_Rs (Sastre et al., 1996). They could also be inhibited by acetylcholine (ACh) through muscarinic type 2 (AChM2) receptors (AChM2Rs; Egan and North, 1986), which have been visualized on GABAergic RFMes neurons (Brischoux et al., 2008). Indeed, elicitation of PS by cholinergic agonists appears to be determined to an important degree by AChM2Rs (Velazquez-Moctezuma et al., 1989; Baghdoyan and Lydic, 1999), which could be associated with inhibition of wake-active GABAergic neurons. Thus it has been suggested that GABAergic neurons of the RFMes could be inhibited through GABA_A_R and/or AChM2R by GABAergic and/or cholinergic PS-active neurons identified in the LDT, SubLDT, and SubC, to permit the occurrence of PS (Sastre et al., 1996; Lu et al., 2006; Brischoux et al., 2008; Boucetta et al., 2014).

Sleep is under homeostatic control, such that extended waking increases the propensity for sleep (Borbély et al., 1984). The activity of individual neurons is also homeostatically regulated (Turrigiano, 1999), whereby prolonged activity results in decreases in excitability and activity through increases in inhibitory and decreases in excitatory receptors (Turrigiano et al., 1998; Kilman et al., 2002; Marty et al., 2004). Previous *in vitro* work on cultured neurons has shown that increased activity of hippocampal neurons results in increases in the density of GABA_A_Rs on their plasma membrane (Marty et al., 2004). Similarly, *in vivo* studies of increased activity in hippocampal neurons induced by seizures showed increases in GABA_A_Rs on the postsynaptic membrane associated with increases in IPSCs (Nusser et al., 1998). It would thus appear possible that presumed wake-active GABAergic RFMes neurons, which would normally control the occurrence of sleep and PS, could undergo homeostatic regulation under conditions of prolonged activity, as would presumably occur in rodents with sleep deprivation (SD) during the day, when they normally sleep the majority of the time.

In this study, we aimed to determine in mice using immunohistochemistry first, by staining for c-Fos, whether RFMes GABAergic neurons were active following SD and second by staining for receptors, whether their prolonged activity was associated with changes in GABA_A_R and/or AChM2Rs in a manner indicative of homeostatic changes.

## Materials and Methods

All procedures conformed to the guidelines of the Canadian council on animal care and were approved by the McGill University Animal Care Committee.

### SD and recovery experimental procedures

A total number of 12 adult male mice (C57BL/6, 20–25 g) were received from the supplier (Charles River) and housed individually under a 12/12 h light/dark cycle (lights on from 7 A.M. to 7 P.M.) at 22°C ambient temperature and with unlimited access to food and water at all times. As described in detail in another manuscript by the authors (Del Cid-Pellitero et al., 2017), animals were maintained in their home cages for the duration of the experiment and recorded by video and telemetric EEG using HomeCageScan software (HomeCageScan 3.0; Clever Systems). For telemetric recording of the EEG, two electrodes were placed symmetrically over parietal cortex along with two for reference over cerebellum and were connected by wires to a transmitter (F20-EET; Data Sciences International, DSI) implanted subcutaneously along the flank. Following surgery, the mice were allowed one week to recover.

The three experimental groups were composed of: (1) sleep control (SC) mice allowed to sleep undisturbed for 2 h from ∼2 to ∼4 P.M. (∼zeitgeber time, ZT 7–9; *n* = 3), (2) sleep deprived mice (SD) maintained awake for 2 h (*n* = 3) or 4 h (*n* = 3) from ∼12 to ∼4 P.M. (∼ZT 5–9), and (3) sleep recovery (SR) mice allowed to sleep for 2 h from ∼2 to ∼4 P.M. (∼ZT 7–9) after being maintained awake for 4 h before euthanasia (*n* = 3). The mice were maintained awake by gentle stimulation with a soft paintbrush of the whiskers each time the mouse appeared to be preparing to sleep. Mice were immediately anaesthetized after the experimental period at ∼4 P.M. (∼ZT 9) with sodium pentobarbital (Euthanyl, 100 mg/kg; Bimeda-MTC) and perfused transcardially with 30 ml of cold saline followed by 200 ml of 3% paraformaldehyde solution. Brains were removed, postfixed in 3% paraformaldehyde for 1 h at 4°C, then placed in 30% sucrose solution at 4°C for 2 d, frozen to −50°C, and stored at −80°C.

Sleep and waking were scored by behavior and EEG using HomeCageScan software.

### Immunohistochemistry

Coronal sections were cut through the brainstem on a freezing microtome at 20-μm thickness and collected in five adjacent series, such that sections were separated by 100-μm intervals in each series. Free floating sections were rinsed in 0.1 M Trizma saline buffer (pH 7.4), then incubated in 6% normal donkey serum buffer for 30 min and subsequently incubated overnight at room temperature in a buffer containing 1% normal donkey serum with combinations of two primary antibodies: mouse anti-GAD67 (1:250, Millipore, catalog #MAB5406, RRID: AB_2278725) or rabbit anti-GABA (1:2000, Sigma-Aldrich, catalog #A2052, RRID: AB_477652) with rabbit anti-c-Fos (1:10,000, Oncogene, Millipore catalog #PC38, RRID: AB_2106755), mouse anti-GABA_A_R β2-3-chain [clone BD17, 1:100, Millipore (Millipore Bioscience Research Reagents), catalog #MAB 341, RRID: AB_2109419], or rabbit anti-AChM2R (1:600, Sigma, catalog #M9558, RRID: AB_260727). Subsequently, sections were incubated at room temperature for 2 h in appropriate combinations of cyanine-conjugated (Cy3 or Cy5) secondary antibodies from donkey (Jackson ImmunoResearch): Cy5-conjugated anti-mouse (1:800, catalog #715-175-150, RRID: AB_2340819) or Cy5-conjugated anti-rabbit (1:800, catalog #711-175-152, RRID: AB_2340607) with Cy3-conjugated anti-rabbit (1:1000, catalog #711-165-152, RRID: AB_2307443) or Cy3-conjugated anti-mouse (1:1000, catalog #715-165-150, RRID: AB_2340813). The GABA_A_R β2-3-chain and AChM2R antibodies were produced and characterized years ago and have since been in use over many years (Levey et al., 1991; Fritschy and Mohler, 1995; Wan et al., 1997; Nusser et al., 1998). GABA and GAD67 antibodies used in this study were proven specific and have been used in previous immunohistochemical studies to identify the GABAergic neurons (Gonchar and Burkhalter, 1997; Brischoux et al., 2008). Sections were subsequently stained with green fluorescent Nissl stain (FNS; 1:2000, N-21480, Invitrogen) for 20 min. Finally, sections were rinsed, mounted and coverslipped with glycerol (Fisher).

### Immunohistochemical image analysis

**Figure 1.**
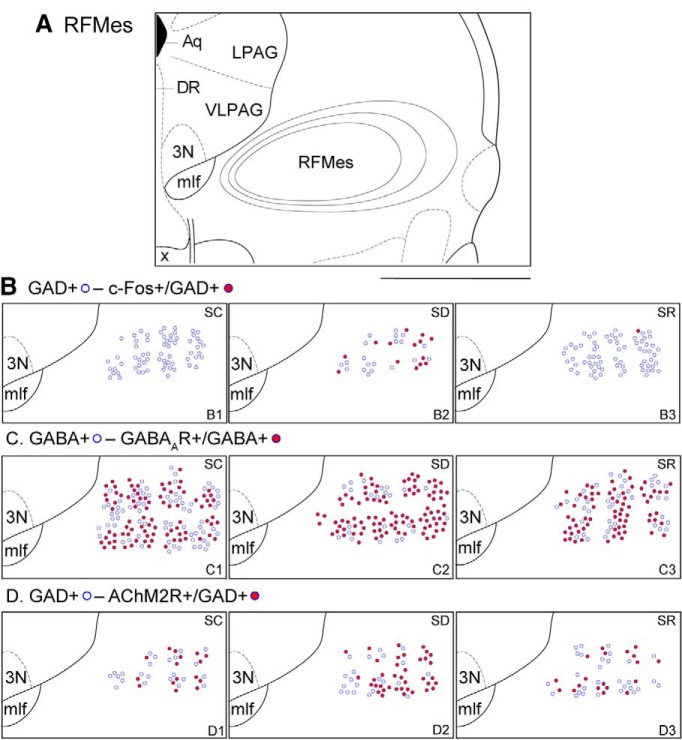
Atlas figure and distribution map of GABAergic neurons expressing c-Fos, GABAARs or AChM2Rs within the RFMes. ***A***, Atlas figure of the pontomesencephalic tegmentum showing the traced contours around the RFMes region in three adjacent sections separated by 100-μm intervals, which were collapsed onto the middle section of this figure (bregma −4.24 mm; Paxinos and Franklin, 2001). ***B***, Distribution map of the GAD+ (blue circle) and c-Fos+/GAD+ (red filled blue circle) neurons in an SC mouse brain (***B1***), an SD mouse brain (***B2***), and an SR mouse brain (***B3***). Note the greater number of c-Fos+/GAD+ neurons in the SD mouse. ***C***, Distribution map of the GABA+ (blue circle) and GABAAR+/GABA+ (red filled blue circle) neurons in an SC mouse brain (***C1***), an SD mouse brain (***C2***), and an SR mouse brain (***C3***). Note the slightly higher number of GABAAR+/GABA+ neurons in the SD mouse. ***D***, Distribution map of the GAD+ (blue circle) and AChM2R+/GAD+ (red filled blue circle) neurons in an SC mouse brain (***D1***), an SD mouse brain (***D2***), and an SR mouse brain (***D3***). Note the greater number of AChM2R+/GAD+ neurons in the SD mouse. Scale bar: 1 mm. 3N, oculomotor nucleus; Aq, aqueduct (Sylvius); DR. dorsal raphe nucleus; mlf, medial longitudinal fasciculus; x, decussation of the superior cerebellar peduncle.

Stained sections were viewed using a Leica DMLB microscope equipped with x/y/z motorized stage, a digital camera (Orca-R^2^, C10600-10B, Hamamatsu photonics K.K.) and fluorescence filters for excitation and emission of Cy2, Cy3, and Cy5 dyes. Images were acquired and analyzed using StereoInvestigator software (MicroBrightField, MBF) and the Optical Fractionator Probe that permit unbiased, systematic random sampling of a region of interest for cell number estimation or measurement of specific parameters, including luminance. Given the application of systematic random sampling for the examination and marking of cells at high magnification and the subsequent measurement of fluorescence intensity of the receptor staining in the marked receptor-positive cells employed here, double blind procedures were not applied in this process. In each series, three sections (at 100-μm intervals) were taken through the RFMes. In each section, a contour was traced under a 5× objective around the RFMes (also called DpMe; Paxinos and Franklin, 2001; Fig. 1*A*). Multi-channel image stacks (with 0.5-μm thickness for each optical section) were acquired under a 40× objective through the mounted histologic section of ∼15-µm thickness. For the purpose of counting and further analysis, a grid size of 300 × 300 μm^2^ and a counting frame of 120 × 120 μm^2^ were used. Across the three sections, ∼21 counting frames were acquired and analyzed per series. Within these images, all cells located below 1 µm from the surface of the section were counted, thus through 14 µm of the section. The average number of GAD-immunostained neurons counted across series on one side was 53.75 ± 3.38 (mean ± SEM), and that of GABA-immunostained neurons was 115.91 ± 8.09, reflecting the different sensitivities of these antibodies or immunostaining for the synthetic enzyme versus the neurotransmitter for GABA. Counting was performed by moving through the z plane to assess the labeling for the c-Fos in the nucleus and for GABA_A_Rs or AChM2Rs on the membrane of the GABAergic neurons within RFMes region. Estimated total numbers of double-labeled cells were computed for each series (c-Fos-GAD, GABA_A_R-GABA, AChM2R-GAD) and expressed as % of estimated GABA+ cell populations per series through the RFMes.

Luminance measurements were also performed in 10 cells per animal for GABA_A_Rs and AChM2Rs on the images, which were acquired under the same gain and exposure using StereoInvestigator (above). They were acquired with the eight-bit setting of the digital camera, which thus provides converted gray scale images of the fluorescence with arbitrary units of 0–256 for luminance measures. Image acquisition was made as rapidly as possible for each cell to avoid bleaching of the fluorescence. As previously described in another article by the authors, a rectangular box sized at 1.5 × 0.3 μm^2^ was placed over the plasma membrane and another box over the nucleus for measurement of background staining in each cell. Subsequently, the luminance intensity of the nucleus was subtracted from that of the membrane in each cell.

Cell counts and luminance measurements were analyzed between experimental groups for GABAergic neurons and c-Fos or different type of receptors (GABA_A_ or AChM2) using one-way ANOVA followed by *post hoc* paired comparisons with Tukey’s HSD correction for difference between groups (SYSTAT Software Inc., version 13). In an initial analysis, the proportion of c-Fos+/GAD+ neurons was found to differ significantly across the original four groups (*F*_(3,8)_ = 9.24, *p* = 0.006); however, due to lack of significant differences between the SD2 and SD4 groups (*post hoc* paired comparisons, *p* = 0.702), they were combined into one SD group in the current report.

Sections were also viewed by LSM 710 confocal laser scanning microscopy equipped with Ar 488-nm, He-Ne 543-nm, and He-Ne 633-nm lasers for excitation and emission of Cy2, Cy3, and Cy5 dyes. Images were acquired under a 63× oil objective with a 1.0 airy unit pinhole size for each channel. All figures were prepared and composed using Adobe Creative Suite (CS4, Adobe System).

## Results

### Sleep-wake states across groups

**Figure 2.**
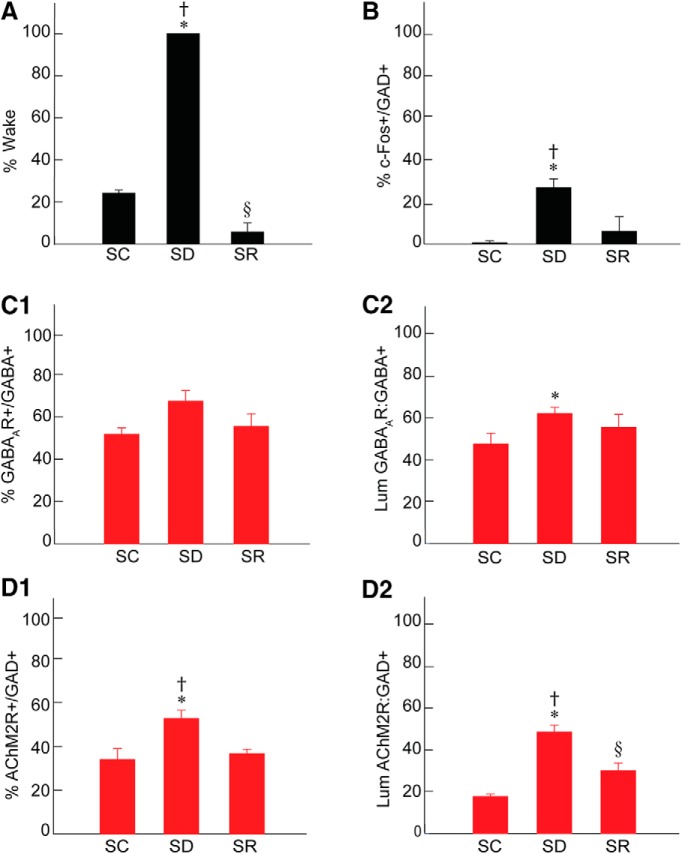
Sleep-wake states, c-Fos, GABA_A_, and AChM2 receptors in RFMes GABAergic neurons across groups. ***A***, The percentage of time spent in wake during the 2 h preceding termination differed significantly across groups, being higher in SD as compared to SC and SR and lower in SR as compared to SC. ***B***, The % of GAD+ neurons that were positively immunostained for c-Fos (+) differed significantly between groups, being greater in SD as compared to SC and SR. ***C***, The % of GABA+ neurons which were positively immunostained for the GABA_A_R (+) increased insignificantly following SD as compared to SC and SR (***C1***). The luminance of the GABA_A_R immunofluorescence on GABA_A_R+/GABA+ neurons differed significantly, being higher in SD as compared to SC (***C2***). ***D***, The % of GAD+ neurons which were positively immunostained for AChM2R (+) differed significantly between groups, being higher in SD as compared to SC and SR (***D1***). The luminance of the AChM2R immunofluorescence on AChM2R+/GAD+ neurons differed significantly, being higher in SD as compared to SC and SR (***D2***). Note that the changes in GABARs and AChM2Rs on RFMes GABAergic neurons parallel the changes in % Wake and % c-Fos+/GAD+ across groups; * indicates significant difference of SD relative to SC; † indicates significant difference of SD relative to SR; § indicates significant difference of SR relative to SC (*p* < 0.05), according to *post hoc* paired comparisons following one-way ANOVA (Table 1).

Mice were prevented from falling asleep in the SD group (*n* = 6) and thereby maintained awake ∼100% of the time, whereas those in the SC group were awake ∼24% of the time (23.79 ± 1.02%, mean ± SEM, *n* = 3) and mice in the SR group ∼6% of the time (5.67 ± 2.63%, *n* = 3) during the 2 h before termination at ∼4 P.M. (Fig. 2*A*; Table 1). Mice in the SR group were awake significantly less than those in the SC group. Being undisturbed, the mice in the SC group thus slept ∼76% of the time and mice in the SR group, which were allowed 2-h recovery after 4-h SD, slept ∼94% of the time, indicating a homeostatic response to SD. The major proportion of time for the SC and SR groups was spent in slow wave sleep (SWS; 66.93 ± 1.71 and 82.29 ± 4.07%, respectively), and a minor proportion in PS (9.28 ± 0.89 and 12.03 ± 0.87%, respectively). Both SWS and PS were significantly increased during SR relative to SC. With regard to behavior, increases in all waking behaviors occurred during SD, but most particularly still behavior, which reflected a quiet waking state (Del Cid-Pellitero et al., 2017).

**Table 1.** Summary of statistics

		One-way ANOVA (group = three levels) *F* value	df (group, error)		Tukey’s HSD paired comparisons *p* value		
Dataset	Figure			*p* value	SC-SD	SC-SR	SD-SR
% Wake	1A	1381.12	2, 9	<0.001	<0.001*	<0.001§	<0.001†
% c-Fos/GAD+	1B	12.97	2, 9	0.002	0.003*	0.708	0.013†
% GABA_A_R+/GABA+	1C1	3.42	2, 9	0.079	0.095	0.880	0.218
Lum GABA_A_R:GABA+	1C2	3.04	2, 117	0.051	0.042*	0.475	0.515
% AChM2R+/GAD+	1D1	8.89	2, 9	0.007	0.013*	0.893	0.028†
Lum AChM2R+:GAD+	1D2	25.36	2, 117	<0.001	<0.001*	0.051§	<0.001†

### c-Fos expression in RFMes GABAergic neurons after SD

**Figure 3.**
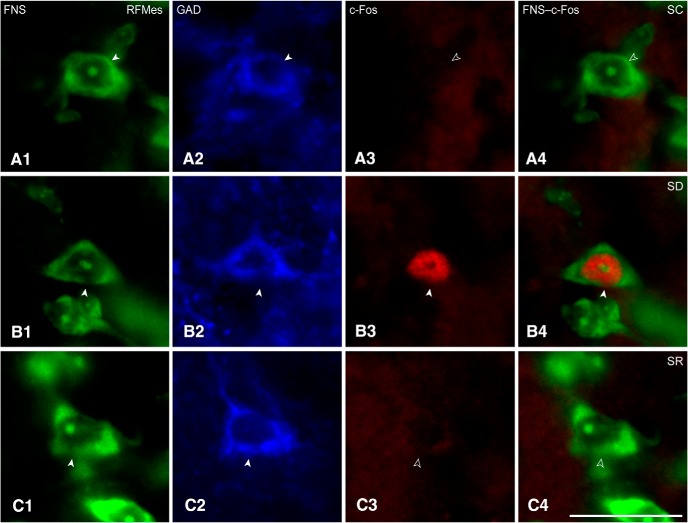
c-Fos in RFMes GABAergic neurons across groups. Fluorescent microscopic images show staining for Nissl with FNS (green; ***A1***, ***B1***, ***C1***), immunostaining for GAD (blue; ***A2***, ***B2***, ***C2***, with positive staining indicated by filled arrowheads), and immunostaining for c-Fos (red; ***A3***, ***B3***, ***C3***, with positive staining indicated by filled arrowhead) along with dual staining for Nissl and c-Fos in merged images (green and red; ***A4***, ***B4***, ***C4***, with positive c-Fos staining indicated by filled arrowhead). Note that c-Fos immunostaining is prominent in the nucleus of a GABAergic neuron from an SD mouse (***B3***, ***B4***), whereas it is not apparent in images from SC or SR mice (***A3***, ***A4*** or ***C3***, ***C4***, indicated by open arrowheads). Scale bars: 20 μm. Image thickness: 500 nm in all panels.

Sections through the RFMes (Fig. 1*A*) were triple-stained for Nissl (FNS), GAD, and c-Fos protein to assess changes in the activity of GABAergic neurons as a function of enforced waking with SD as compared to SC and SR (Fig. 3*A–C*). c-Fos immunostaining was relatively rarely seen in the GAD+ neurons of the SC or SR mice, whereas it was prominent in the nucleus of multiple GAD+ neurons of the SD mice (Figs. 1*B1–B3* , 3*A–C*). The average proportion of c-Fos+/GAD+ cells differed significantly between groups (*n* = 3 or 6 mice per group; Fig. 2*B*; Table 1), being significantly greater in SD (26.93 ± 3.41%) as compared to SC (0.66 ± 0.54%) and SR (6.02 ± 4.35%) groups. The % c-Fos+/GAD+ cells did not differ between SC and SR indicating that the proportion of c-Fos+/GAD+ neurons, returned to control or baseline levels following SR.

### GABA_A_Rs on RFMes GABAergic neurons after SD

**Figure 4.**
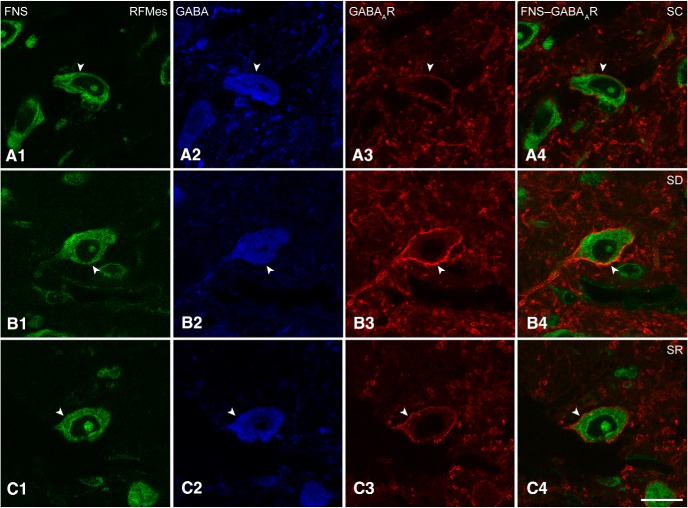
GABA_A_Rs in RFMes GABAergic neurons across groups. Confocal microscopic images show all neurons stained for Nissl with FNS (green; ***A1***, ***B1***, ***C1***), the GABAergic neurons immunostained for GABA (blue; ***A2***, ***B2***, ***C2***, indicated by filled arrowhead), and for the GABA_A_Rs in single (red; ***A3***, ***B3***, ***C3***, indicated by filled arrowhead) and merged images (***A4***, ***B4***, ***C4***, indicated by filled arrowhead**).** Note that in an SC mouse, the GABA_A_R immunofluorescence is minimally visible, whereas in an SD mouse, it is prominent and bright. In an SR mouse, it appeared less bright than in the SD mouse. In all cases, the immunostaining is relatively continuous though with nonuniform intensity along the plasma membrane of the GABA+ neurons. Scale bars: 20 μm. Image thickness: 500 nm in all panels.

Triple-stained sections for FNS-GABA-GABA_A_R were analyzed to assess the presence and intensity of GABA_A_Rs on RFMes GABAergic neurons in brains of mice from SC, SD, and SR groups (Fig. 4*A–C*). GABA_A_R immunostaining appeared to be primarily located on the plasma membrane of the GABA+ neurons. It varied in intensity among neurons and mice yet appeared to be consistently most intense in GABA+ neurons of the SD mice (Fig. 4*A–C*).

A major proportion of the GABA+ neurons in the RFMes appeared to be positively immunostained (+) for the GABA_A_R over the plasma membrane in all mice of all groups. However, the proportion of GABA_A_R+/GABA+ neurons appeared to be somewhat greater in the SD group as compared to SC and SR groups (Fig. 1*C1–C3* ). The average proportion of GABA_A_R+/GABA+ neurons differed by a trend between groups (*n* = 3 or 6 mice per group; Fig. 2*C1* ; Table 1) and was increased by a trend following SD (67.33 ± 4.14%) as compared to SC (51.75 ± 1.84%) but not as compared to SR (55.42 ± 3.69%). The % GABA_A_R+/GABA+ neurons also did not differ between SC and SR indicating that the proportion of GABA_A_R+/GABA+ neurons returned toward control or baseline levels following SR.

The luminance of the GABA_A_R immunostaining on the plasma membrane of GABA_A_R+/GABA+ cells was significantly different between groups (*n* = 30 or 60 cells per group; Fig. 2*C2*; Table 1), being significantly higher in SD (61.96 ± 2.85) as compared to SC (47.35 ± 5.01) but not different as compared to SR (55.39 ± 5.89). The luminance did not differ between SC and SR indicating that the density of GABA_A_Rs on the membrane of the GABA+ neurons returned to control or baseline levels following SR.

### AChM2Rs on RFMes GABAergic neurons after SD

**Figure 5.**
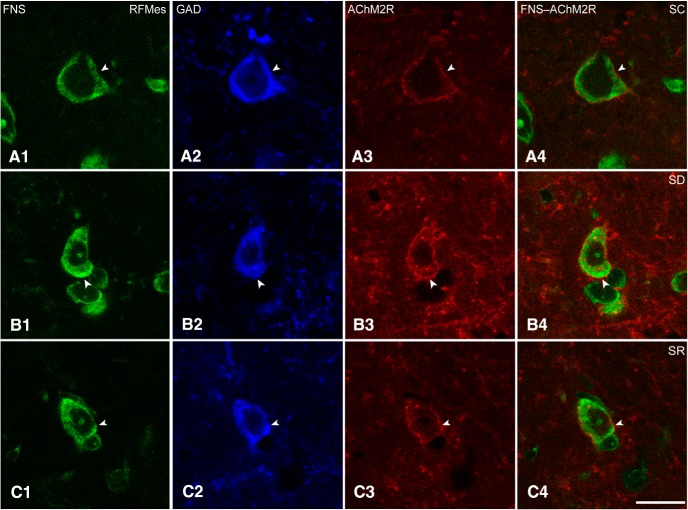
AChM2Rs in RFMes GABAergic neurons across groups. Confocal microscopic images show all neurons stained for Nissl with FNS (green; ***A1***, ***B1***, ***C1***), the GABAergic neurons immunostained for GAD (blue; ***A2***, ***B2***, ***C2***, indicated by filled arrowhead), and for the AChM2Rs in single (red; ***A3***, ***B3***, ***C3***, indicated by filled arrowhead), and merged images (***A4***, ***B4***, ***C4***, indicated by filled arrowhead). Note that in an SC mouse, the AChM2R immunofluorescence is minimally visible along the plasma membrane, whereas in an SD mouse, the AChM2R staining is bright and clearly visible along the full membrane of the GAD+ neuron. In an SR mouse, the staining appeared less bright than in the SD mouse. Scale bars: 20 μm. Image thickness: 500 nm in all panels.

Sections triple-stained for FNS-GAD-AChM2R were analyzed to assess the presence and intensity of AChM2Rs on RFMes GABAergic neurons in brains of mice from SC, SD and SR groups (Fig. 5*A–C*). The AChM2R immunostaining was observed over the plasma membrane of the GAD+ neurons of all groups, although it appeared to be the most prevalent and dense over neurons of the SD group (Fig. 5*A–C*).

The proportion of AChM2R+/GAD+ neurons in the RFMes appeared to be somewhat higher in the SD group as compared to the SC and SR groups (Fig. 1*D1–D3* ). The average proportion of the AChM2R+/GAD+ neurons differed significantly between groups (*n* = 3 or 6 mice per group; Fig. 2*D1*; Table 1), being significantly higher following SD (52.63 ± 3.17%) compared to SC (34.00 ± 3.05%) and SR (36.66 ± 1.21%). The % AChM2R+/GAD+ did not differ between SC and SR indicating that proportion of AChM2R+/GAD+ neurons returned to control or baseline levels following SR.

The luminance of the AChM2R immunofluorescence over the membrane of the AChM2R+/GAD+ cells was significantly different between groups (*n* = 30 or 60 cells per group; Fig. 2*D2*; Table 1), being significantly greater in the SD (48.40 ± 3.27) as compared to SC (17.65 ± 1.15) and SR groups (29.91 ± 2.84). The luminance measure was also significantly higher in SR as compared to SC indicating that the density of AChM2Rs on the membrane of the GAD+ neurons did not fully return to the baseline control level with SR.

## Discussion

The present results show that RFMes GABAergic neurons express c-Fos with SD and are thus likely more active during enforced waking during the day, when rodents normally sleep the majority of the time. Immunohistochemical images also show increases in GABA_A_R and AChM2R on the RFMes GABAergic neurons with SD, suggesting that with increased activity during enforced waking, the neurons are regulated in a homeostatic manner by increases in receptors which elicit inhibition.

### c-Fos expression in RFMes GABAergic neurons following SD

In triple-stained sections through the RFMes, c-Fos immunostaining was evident in the nucleus of multiple GAD+ neurons in the SD group, whereas it was seen in few or no GAD+ neurons in the SC and SR groups. Somewhat different results were reported in rats following selective and prolonged (72 h) PS deprivation, which was associated with increased c-Fos in VLPAG/DpMes GAD+ neurons with both SD and SR as compared to SC (Sapin et al., 2009). Here using total and short (2–4 h) SD, the average proportion of c-Fos+/GAD+ neurons significantly increased only following SD as compared to SC and returned to control levels with SR. Similar increases in c-Fos expression have been documented following SD as compared to SC and SR in orexin (Orx) neurons, which from electrophysiological recordings, are known to discharge during waking and to be silent during sleep (Lee et al., 2005; Modirrousta et al., 2005). In contrast, significantly less c-Fos expression was found following SD as compared to SR in melanin concentrating hormone (MCH) neurons, which are known from recordings to be silent during waking and discharge during sleep (Modirrousta et al., 2005; Hassani et al., 2009). We thus interpret our results to suggest that RFMes GABAergic neurons are active during enforced waking with SD and possibly not active or less active during SWS and PS with SC and SR. However, such assumptions can only be proven by electrophysiological recordings of identified GABAergic neurons in the RFMes.

### Homeostatic regulation of RFMes GABAergic neurons through GABA_A_Rs

In triple-stained sections, GABA_A_Rs were apparent over the plasma membrane in a relatively large proportion of RFMes GABA+ neurons, which was the greatest following enforced waking with SD. The fluorescence intensity of the GABA_A_Rs was significantly higher on the membrane of the GABA+ neurons following enforced waking and presumed increased activity with SD as compared to SC or SR. The increases in GABA_A_Rs on RFMes neurons are similar to those seen on Orx and motor 5 (Mo5) neurons following enforced waking with SD, when these neurons have also been shown to be active according to c-Fos expression and/or electrophysiological recordings (Toossi et al., 2016, 2017). They are also similar to increases in GABA_A_Rs seen on cholinergic basal forebrain neurons and cortical excitatory neurons following enforced waking and cortical activation with SD (Modirrousta et al., 2007; Del Cid-Pellitero et al., 2017). Collectively, these studies suggest that many cell groups which are normally active during waking and in a relative resting state during sleep undergo homeostatic regulation when submitted to enforced waking during the period when rodents normally sleep the majority of the time. Homeostatic changes associated with sleep and waking would include changes in glutamate as well as GABARs, which have been documented in the cortex and for which hypotheses and conclusions concerning Hebbian synaptic plasticity versus homeostatic synaptic scaling have diverged among scientists, and results have differed depending on the specific experimental paradigms and techniques employed (Tononi and Cirelli, 2003; Vyazovskiy et al., 2008; Aton et al., 2009; Xie et al., 2016; Del Cid-Pellitero et al., 2017; Diering et al., 2017; Puentes-Mestril and Aton, 2017; Timofeev and Chauvette, 2017).

Changes in GABA_A_Rs seen here can be likened to those seen in *in vitro* and *in vivo* studies, in which increased activity induced pharmacologically or by seizures resulted in increased GABA_A_Rs on the plasma membrane and correlated increases in IPSCs (Nusser et al., 1998; Marty et al., 2004). Such homeostatic changes in receptors have been shown to occur within short time periods (Yamanaka et al., 2000; Ibata et al., 2008; Xie et al., 2016), similar to the period of SD employed here. The GABA_A_Rs on the membrane of the RFMes GABA+ neurons returned to control levels here during increased sleep with SR, when according to the lack of c-Fos expression, the neurons were likely less active or inactive. Similar decreases of GABA_A_Rs on the membrane occurred with SR in Orx neurons, when these neurons are known to be silent (Lee et al., 2005), whereas increases of GABA_A_Rs occurred with SR on MCH neurons, when these neurons are known to discharge (Hassani et al., 2009; Toossi et al., 2016). Such changes can be likened to decreases in GABA_A_R clusters on the membrane of cultured neurons after pharmacologically induced periods of inactivity, which are associated with decreases in IPSCs (Kilman et al., 2002; Marty et al., 2004). The current results thus suggest homeostatic regulation through the GABA_A_Rs that would be associated with decreases in excitability following prolonged activity with SD and restorative return to baseline excitability following inactivity with SR.

Pharmacological evidence suggests that GABAergic neurons within the VLPAG and adjacent region of RFMes (or DpMe), which project to the pontine tegmentum, could be responsible for holding PS-effector neurons under inhibition during waking (Sastre et al., 1996; Xi et al., 1999; Boissard et al., 2002; Sanford et al., 2003). This suggestion was recently confirmed in an optogenetic study showing that selective activation of GABAergic neurons within the VLPAG region suppressed the onset of PS or shortened the duration of PS episodes (Weber et al., 2015). Optogenetic evidence was also presented to indicate that GABAergic neurons in the ventral medulla discharge during PS and could inhibit the presumed wake-active VLPAG GABAergic neurons to promote PS (Weber et al., 2015). GABAergic neurons have also been recorded in the LDT/SubLDT and adjoining medial pedunculopontine tegmental (PPT) nuclei, which discharge maximally during PS and could thus possibly participate in the inhibition of GABA_A_R-bearing RFMes GABAergic neurons during PS (Boucetta et al., 2014). In addition, there are PS-active GABAergic neurons in the hypothalamus which project into the VLPAG/RFMes region and appear to inhibit GABAergic wake-active neurons therein (Hassani et al., 2010; Clément et al., 2012). Some of these PS-active GABAergic neurons, which project to the VLPAG/RFMes region, also contain and use MCH (Hassani et al., 2009; Hassani et al., 2010; Clément et al., 2012). MCH neurons have been shown in electrophysiological recordings to discharge during sleep and PS (Hassani et al., 2009) and in optogenetic studies to enhance sleep with PS when stimulated, in part through release of GABA (Jego et al., 2013; Konadhode et al., 2013; Tsunematsu et al., 2014).

Here, we present evidence for the first time that presumed wake-active GABAergic neurons in the RFMes could undergo homeostatic plasticity with decreased excitability following increased activity with SD through increases in GABA_A_Rs over their plasma membranes. They would accordingly be more sensitive to inhibitory input from sleep-active GABAergic neurons of the pons, medulla or hypothalamus and thus susceptible to permitting the onset of sleep following SD or deficiency.

### Homeostatic regulation of RFMes GABAergic neurons through AChM2Rs

Similar to the distribution of GABA_A_Rs, AChM2Rs were apparent over the plasma membrane in a proportion of RFMes GAD+ neurons across groups. Following SD, the proportion and luminance of the AChM2Rs on the plasma membrane of the RFMes GAD+ neurons increased relative to SC, presumably due to prolonged activity of RFMes GABAergic neurons during enforced waking. The proportion and density of the AChM2Rs on GAD+ neurons returned to control level with SR, presumably due to less activity or inactivity of the GABAergic neurons following SR. Our results here are parallel to the increase in proportion and density of AChM2Rs seen on the membrane of the Mo5 neurons following SD with prolonged waking, when these neurons are also presumed to be active (Toossi et al., 2017).

It was shown in pharmacological studies that microinjection of carbachol into the pontomesencephalic tegmentum, though most effectively into the pontine tegmentum, elicits PS with muscle atonia (George et al., 1964; Gnadt and Pegram, 1986; Baghdoyan et al., 1987; Vanni-Mercier et al., 1989). The induction of PS by administration of cholinergic agonists delivered into the pontomesencephalic tegmentum is dependent on AChM2Rs (Velazquez-Moctezuma et al., 1989; Baghdoyan and Lydic, 1999), which mediate hyperpolarization and inhibition (Egan and North, 1986). It can thus be posited that neurons which normally inhibit PS likely express AChM2Rs on their membrane. Given the immunohistochemical evidence for the presence of AChM2Rs on the membrane of RFMes GABAergic neurons (Brischoux et al., 2008), it is suggested that ACh could inhibit RFMes GABAergic neurons through their AChM2Rs. Juxtacellular recordings and labeling of cholinergic neurons within the LDT/SubLDT/PPT show that they are maximally active during wake and PS (Boucetta et al., 2014). Thus, cholinergic neurons could exert an inhibitory influence on neurons through AChM2Rs during PS, but would also do so during waking. Yet some of these GABAergic RFMes neurons were found to also bear Orx2Rs (Brischoux et al., 2008), and selective lesions of the Orx2R-bearing GABAergic neurons in this region were found to increase PS (Kaur et al., 2009). The AChM2Rs on the RFMes GABAergic neurons could normally be in balance with Orx2Rs, such that the excitatory action of Orx on the GABAergic neurons would normally prevail during waking, when Orx neurons discharge (Lee et al., 2005), whereas the inhibitory action of ACh would prevail in the absence of Orx, as during sleep and PS in normal cases or during narcolepsy with cataplexy in abnormal cases (Nishino and Mignot, 1997, 2011). With an increase of AChM2Rs on the GABAergic neurons with SD shown here, the inhibitory action of ACh could possibly also prevail during waking. Inhibition of RFMes GABAergic neurons by ACh would remove the GABAergic inhibition imposed by these neurons on PS-effector neurons located within the pontine tegmentum and permit the occurrence of PS or possibly increase the probability of narcolepsy with cataplexy, as occurs in patients under conditions of sleep deficits (Nishino and Mignot, 1997, 2011). Here, we present evidence that RFMes GABAergic neurons could undergo homeostatic plasticity with decreases in excitability following increased activity with SD through increases in AChM2Rs and resulting increased susceptibility to permitting sleep and PS with muscle atonia.

We conclude that RFMes GABAergic neurons are homeostatically regulated across the sleep-waking cycle through GABA_A_R and AChM2R. The density of these receptors increases on the membrane of RFMes GABAergic neurons due to increased activity during enforced waking with SD, which would result in increased responsiveness of the neurons to inhibition by GABA and ACh. Their density is restored to baseline levels with SR when the neurons are likely inactive, which would result in the return of excitability and activity of the RFMes GABAergic neurons to stable levels. The homeostatic regulation of these presumed wake-active, PS-gating RFMes GABAergic neurons would in turn be paralleled by the similar regulation of their afferent wake-active Orx neurons, which would normally excite them, and the inverse regulation of their afferent PS-active MCH/GABAergic neurons, which would normally inhibit them (Toossi et al., 2016). The homeostatic changes in the different sleep-wake regulatory neurons and circuits (Jones, 2017) likely underlie the homeostatic regulation of sleep-wake states.

## Synthesis

Reviewing Editor: Rae Silver, Columbia University Decisions are customarily a result of the Reviewing Editor and the peer reviewers coming together and discussing their recommendations until a consensus is reached. When revisions are invited, a fact-based synthesis statement explaining their decision and outlining what is needed to prepare a revision will be listed below. The following reviewer(s) agreed to reveal their identity: Chiara Cirelli, John Peever

This is an interesting study and demanding study that has been carefully executed. The findings are novel and important not only to the field of sleep biology, but also to the field of homeostatic plasticity - a key aspect of the work. The authors describe interesting changes in the expression of GABAergic and muscarinic M2 receptors (Rs) on inhibitory neurons located in the VLPAG and RFMes regions after a few (2-4) hours of total sleep deprivation. In their model, the VLPAG and RFMes regions inhibit, during waking, the PS/REM-on area of the pons, and are in turn under the inhibitory control of cholinergic and GABAergic neurons in the pons.

The authors interpret these results as “homeostatic” changes due to prolonged neuronal activity of the GABAergic cells in the VLPAG and RFMes regions, which would be active during total sleep deprivation and need to be strongly inhibited. This interpretation, however, seems to rely on the assumption that 2-4 hours of waking leads to a sustained increase in neuronal activity, significant enough to trigger homeostatic changes as those described by Turrigiano and colleagues. However, there seems to be no direct evidence for a sustained increase in neuronal activity; the data show a change in Fos expression, but Fos induction does not necessarily reflect sustained (as opposed to phasic and short lasting) increase in activity. Moreover, the references cited for homeostatic scaling (Turrigiano, Kilman, Marty) refer to studies in which a profound suppression of neuronal activity was induced in vitro, and lasted several days. It is not clear whether the same kind of changes can happen in just a few hours of physiological activity during waking (no seizures, no total block of activity) remains to be demonstrated. The authors should acknowledge the lack of direct evidence.

The experiments do not provide direct evidence that the increase or trend increase in GABAergic and muscarinic M2 Rs actually occurs in the Fos positive neurons; this is likely due to technical issues, but ideally, the authors should provide this evidence or clearly discuss this limitation. It is unclear how the changes seen can be interpreted as a response to sustained activity if they also occur in Fos negative cells. As the authors acknowledge, the VLPAG and RFMes regions contain not only REM/PS off neurons, but also many REM/PS on neurons. In theory, the Rs changes should be limited to the first group of cells, but this is not shown. The authors also do not show that the changes are due to “scaling; i.e. they are multiplicative in nature. (It should be acknowledged, perhaps in the discussion, that Rs expression as measured in this study is an indirect measure, and its functional significance will need to be supported by future, more functional studies.)

The authors frame their results as homeostatic changes as described by Turrigiano, rather than the results by the Tononi/Cirelli, Tafti, and Huganir groups about scaling of glutamatergic receptors in cortex and forebrain due to sleep and waking. One could interpret the current results as an indication that the strength of some midbrain GABAergic and cholinergic synapses also increases with waking. This uncertainty can be resolved by making the discussion less speculative and more inclusive of other relevant studies.

In summary, the limitations in the interpretation of the results need to be clearly acknowledged. The authors must refrain from conclusions that are overly speculative. The alternative is to perform more experiments - though these would most likely take more than 2 months.

Specific comments:

-The abstract is very technical and overly focused for a “non-sleep” audience. The significance statement is far clearer. I suggest the authors broaden and simplify their language in the abstract so that the naïve, non-sleep expert, can more easily absorb the findings and importance of this excellent study.

- In terms of immunohistochemical (imaging) analysis (cFos, receptor staining) it is unclear if the authors were “blinded” to which group (i.e., SR, SC, SR) they were analyzing. It would be useful to indicate if the authors were blinded to these conditions.

- In Fig. 2 the authors plot amounts of wakefulness, but in some respects PS amounts are another important variable of interest. Authors might also provide data showing how much PS (and SWS) each group experienced. While it is clear that amounts of waking varied among the SD, SR and SC groups, it would be helpful to understand if PS amounts differed between the SR and SC groups. As it stands it's difficult to determine if there was more PS in the SR group than in the SC group. And this is an important point given that the authors indicate (page 12, line 214) that there is a homeostatic response to sleep deprivation.

- The authors interpret their data from the perspective of wake and sleep amounts, however, an alternative possibility is that changes in motor activity (associated with enforced wakefulness) is the driving force as opposed to sleep/wakefulness. The authors might consider adding a sentence or two to the discussion section to address this possibility.

- Page 5, line 74. The word “prominent” seems out of place.

- Page 6, lines 89-91. This sentence is awkward.

- Page 12, line 225-266. It is unclear what is meant by “(n=3 to 6 mice per group)". Do you mean there are 3 groups with 6 mice in each group?
